# Supplementary material for: TMPRSS11E-mediated TFR1 cleavage influences IFN-γR2 internalization and the macrophage innate response
Source: Commun Biol. 2025 Dec 3;8:1741. doi: 10.1038/s42003-025-09132-2 (PMC12675585; doi:10.1038/s42003-025-09132-2)

Supplementary Information

Supplementary Table 1. Plasmids and antibodies

Name	Source	Identifier
**Plasmids**		
pCMV-V5-TFR1-myc	This paper	N/A
pCMV-V5-TFR1-myc mut1	This paper	N/A
pCMV-V5-TFR1-myc mut2	This paper	N/A
pCMV-GFP	Beyotime	D2707
pCMV-GFP-TMPESS11E	This paper	N/A
pCMV-GFP-TMPESS11E S372A	This paper	N/A
pET-32a-TMPRSS11E	This paper	N/A
pCMV-TMPRSS11E-Flag	This paper	N/A
pLV3-CMV-TMPRSS11E- Flag-Puro	This paper	N/A
pLV3-CMV-TMPRSS11E S372A-Flag-Puro	This paper	N/A
pCDH-CMV-MCS-EF1-CopGFP-T2A-puro	Addgene	Cat#72263
pLP1	Miaolingbio	P0264
pLP2	Miaolingbio	P0265
pLP/VSVG	Miaolingbio	P0266
**Antibodies**		
Anti-TFR1(WB/ICC)	Abcam	ab269513
Anti-IFN-γR2(WB/IF)	Abcam	ab171081
Anti-iNOS(WB)	Abcam	ab283655
Anti-TMPRSS11E(WB)	Thermos fisher scientific	PA548775
Anti-TMPRSS11E(IHC/IF/ICC)	Thermos fisher scientific	PA550809
Anti-CD68(IHC)	Proteintech company	28058-1-AP
Anti-CD86(FC)	Proteintech company	13395-1-AP
Anti-CD163(FC)	Proteintech company	16646-1-AP
Anti-STAT1(WB)	Proteintech company	10144-2-AP
Anti-pSTAT1(WB)	Proteintech company	28979-1-AP
Anti-RAB5a(IF)	Proteintech company	66339-1-Ig
Anti-α-Tubulin (WB)	Proteintech company	66031-1-Ig
Anti-GFP (WB)	Proteintech company	66002-1-Ig
Anti-Flag (WB/IP)	Proteintech company	66008-4-Ig
Anti-myc (WB)	Proteintech company	16286-1-AP
Anti-V5 (WB/IP)	Proteintech company	14440-1-AP
Mouse lgG	Beyotime	A7028
Rabbit lgG	Beyotime	A7016
HRP-labeled Goat Anti-Rabbit IgG	Beyotime	A0208
HRP-labeled Goat Anti-Mouse IgG	Beyotime	A0216
Anti-GAPDH(WB)	Proteintech company	60004-1-Ig

Supplementary Table 2. Chemicals

REAGENT	SOURCE	IDENTIFIER
**Chemicals**		
GenEscortII	Nanjing Wisegen Biotechnology	Cat# WIS 2100
Mut Express™ II Fast Mutagenesis Kit	Vazyme	Cat# C214-01
Lipopolysaccharides from Escherichia coli O55:B5	Beyotime	Cat# ST1470
Super ECL Detection Reagent	YEASEN	Cat# 36208ES60
Triton X-100	Bio-Rad	Cat# 161-0407
Immun-Blot PVDF Membrane	Bio-Rad	Cat# 162-0177
RIPA Lysis buffer	Beyotime	Cat# P0013B
BSA	Beyotime	Cat# ST025
Paraformaldehyde	Sangon Biotech	Cat# A500684
BCA Protein Assay Kit	Thermo Fisher Scientific	Cat# 23227
Protein A+G Agarose	Beyotime	Protein A+G Agarose
Human TFRC protein (Fc-TFR1)	Sino biotechnology	11020-H01H
IPTG	Sangon biotech	A600168
Hematoxylin and Eosin Staining Kit	YEASEN	60524ES60
DAB chromogenic kit	MXB Biotechnology	DAB-0031
Double-labeled multiplex immunofluorescence kit	AiFang biological	AFIHC023
Camostat Meslate	Solarbio life sciences	IC2310
Phorbol 12-myristate 13-acetate	MedChemExpress	HY-18739
IFN-γ	novoprotein	C014
IL-4	novoprotein	C050
Alexa-488-conjugated Tf	Thermos fisher scientific	T13342
HisPurTM Ni-NTA Resin	ThermoFisher Scientific	88221
sTFR1 ELISA kit	Cusabio	CSB-E08389m
Myeloperoxidase (MPO) activity assay kit	Sigma-Aldrich	MAK068
Plasma Membrane Protein Isolation kit	Invent Biotechnologies	SM -005
Endosome Isolation and Cell Fractionation kit	Invent Biotechnologies	ED-028

Supplementary Table 3. Characteristics of patients with infectious pneumonia

Characteristics	Patients with infectious pneumonia	Control patients with pulmonary nodules
Gender (Male/Female)	15/5	6/4
Age range(median)	30-68 (45)	26-65(41)
Sample size	20	10

Supplementary Figure 1

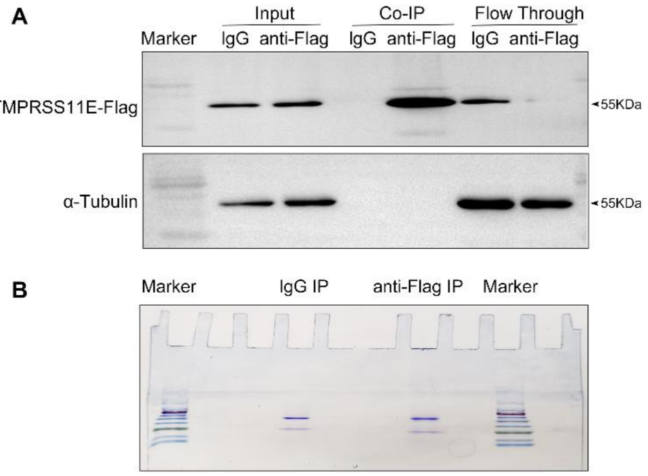

Supplementary Figure 1. Co-IP and preparation of immunoprecipitated protein samples for LC-MS/MS. A. pCMV-TMPRSS11E-Flag was transfected into HEK293T cells. Cell lysates were immunoprecipitated with anti-Flag antibody or control IgG. Pull-down efficiency of TMPRSS11E-Flag protein by anti-Flag was confirmed by western. **B.** Preparation of protein samples for LC-MS/MS. The co-immunoprecipitated samples were obtained and then run short-distance SDS-PAGE gels. When sample proteins migrated 5mm into resolving gel, stop electrophoresing. The gels were stained with Coomassie brilliant blue. Protein size markers were indicated (MW). The lane of stained proteins were excised for in-gel digestion and subsequent LC-MS/MS. Note that leaving free lanes between samples to avoid potential cross-contamination that can occur if the wells are overloaded. A short gel separation is desirable for this application as this minimizes the total gel volume in the in-gel digestion and increases depth of analysis.

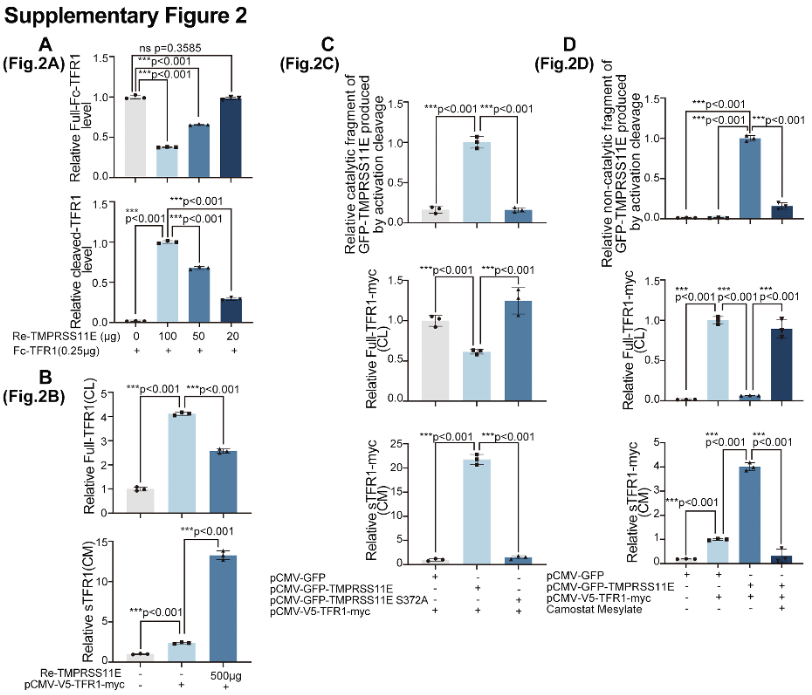

Supplementary Figure 2. Statistical analysis of protein levels in Fig.2A, Fig.2B, Fig.2C and Fig.2D. Data are expressed as mean ± SD (n =3), *p < 0.05, **p < 0.01. ***p < 0.001, ns, not significant. A. Quantitation of protein expressions in (Fig.2A) (n = 3). B. Quantitation of protein expressions in (Fig.2B) (n = 3). C. Quantitation of protein expressions in (Fig.2C) (n = 3). D. Quantitation of protein expressions in (Fig.2D) (n = 3).

Supplementary Figure 3

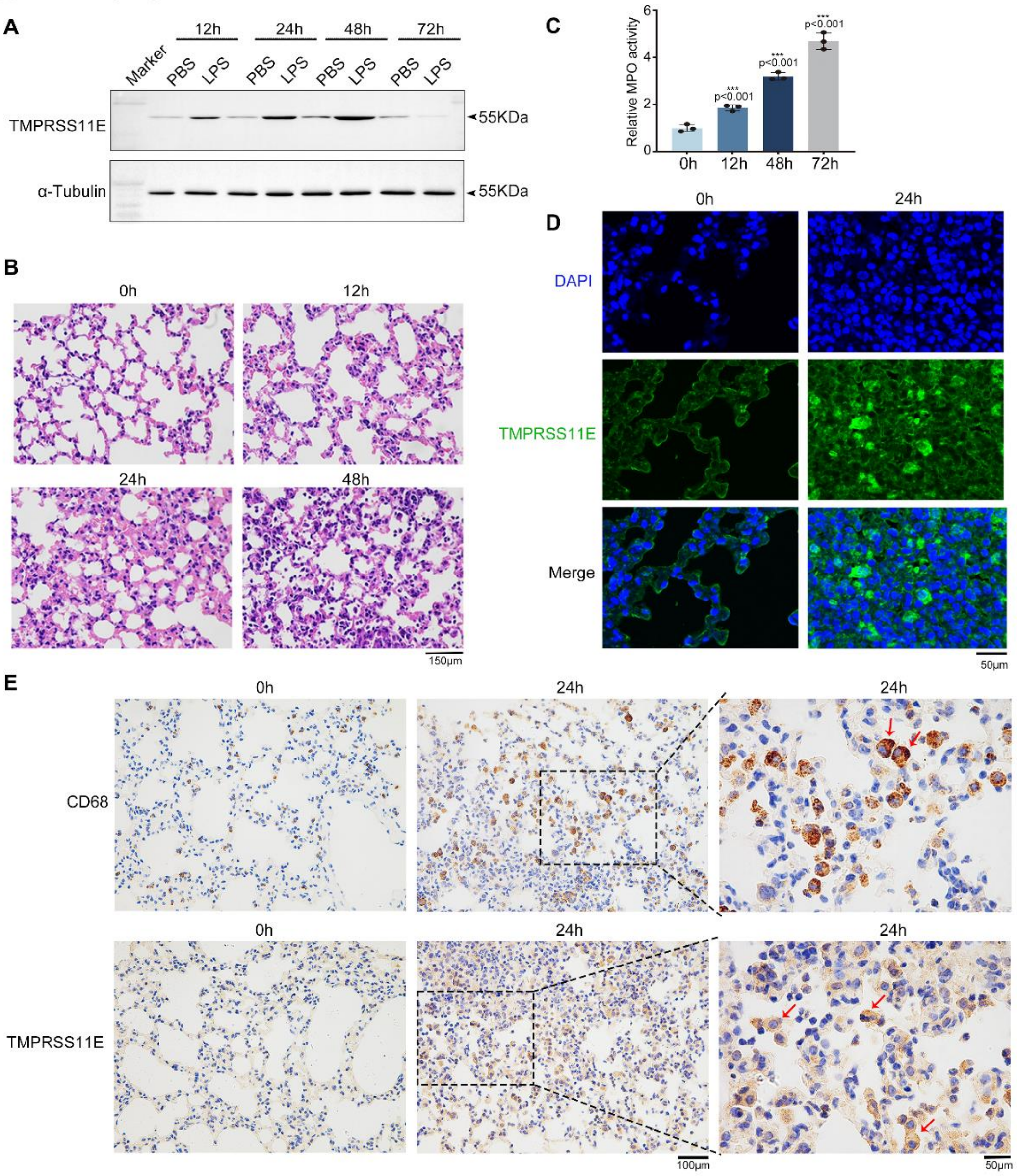

Supplementary Figure 3. TMPRSS11E highly expressed in alveolar macrophage of LPS stimulated mice. A. Western blotting analysis of TMPRSS11E in the whole-lung homogenates with LPS stimulated. **B.** Pathological changes in lung tissues were analyzed by hematoxylin and eosin staining. Scale bars, 150μm. **C.** Measurement of MPO in lung tissues. Lung tissues were homogenized in lysis buffer, and then centrifuged to collect the supernatants. Myeloperoxidase (MPO) activity was measured according to the manufacturer's instructions. D. Immunofluorescence staining with TMPRSS11E in the lung tissues of LPS challenged mice. Scale bars, 50μm. E. Immunohistochemistry of lung sections stained with TMPRSS11E antibody or CD68 antibody and counterstained with hematoxylin. Red arrows indicated macrophages. Scale bars on the left, 100μm; scale bars on the right, 50μm. (n=3 for each group).

Supplementary Figure 4

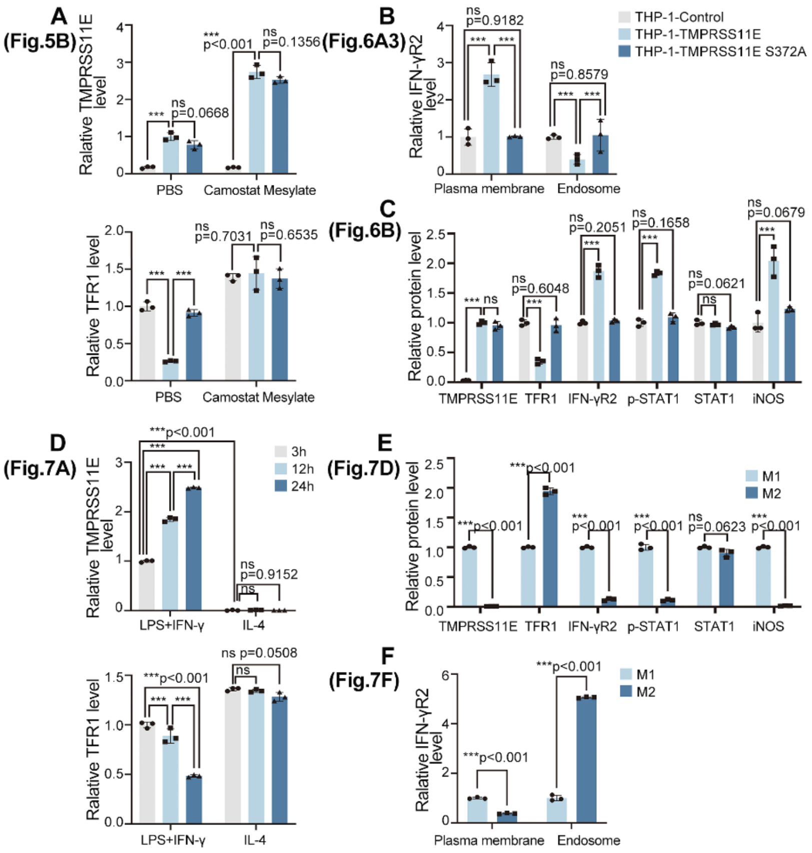

Supplementary Figure 4. Statistical analysis of protein levels in Fig.5B, Fig.6A3, Fig.6B, Fig.7A, Fig.7D and Fig.7F. Data are expressed as mean ± SD (n =3), *p < 0.05, **p < 0.01. ***p < 0.001, ns, not significant. A. Quantitation of protein expressions in (Fig.5B) (n = 3). B. Quantitation of protein expressions in (Fig.6A3) (n = 3). C. Quantitation of protein expressions in (Fig.6B) (n = 3). D. Quantitation of protein expressions in (Fig.7A) (n = 3). E. Quantitation of protein expressions in (Fig.7D) (n = 3). F. Quantitation of protein expressions in (Fig.7F) (n = 3).

Supplementary Figure 5

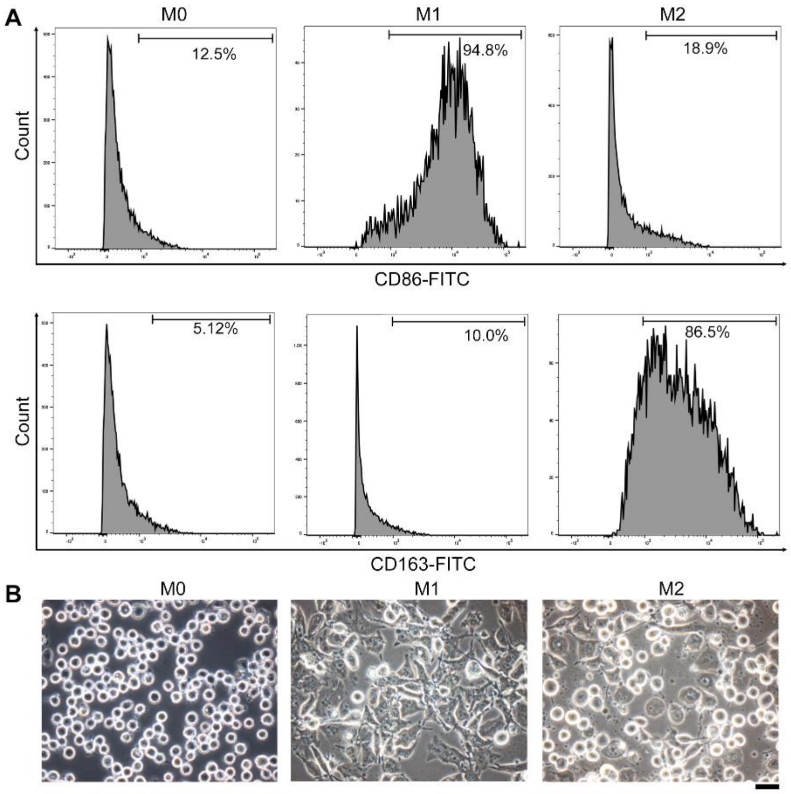

Supplementary Figure 5. Characterization of the human THP-1 monocyte differentiated macrophages. THP-1 cells were cultured in 200 ng/ml phorbol-12-myristate-13-acetate (PMA) for 24 hours to establish M0. M0 cells were cultured in 100 ng/ml LPS and 100 ng/ml IFN-γ for 24 hours to establish M1. M0 cells were cultured in 20 ng/ml IL-4 for 24 hours to establish M2. **A.** Use the blank to set the voltage, then select the main cell group, choose the gating and then select FSC/SSC gates to remove debris, use Single Parameter Histograms to show the percentage of positive-stained cells. Macrophage surface markers CD86 (M1 marker) and CD163 (M2 marker) in THP-1-derived macrophage were measured by flow cytometry. Representative results are shown. **B.** Cell morphology of macrophages subtypes derived from human THP-1 cells was observed under light microscope. Representative image is shown. Scale bars, 50μm. (n=3 for each group).

Supplementary Figure 6. Unprocessed original images of western blots for figures

Figure 1D

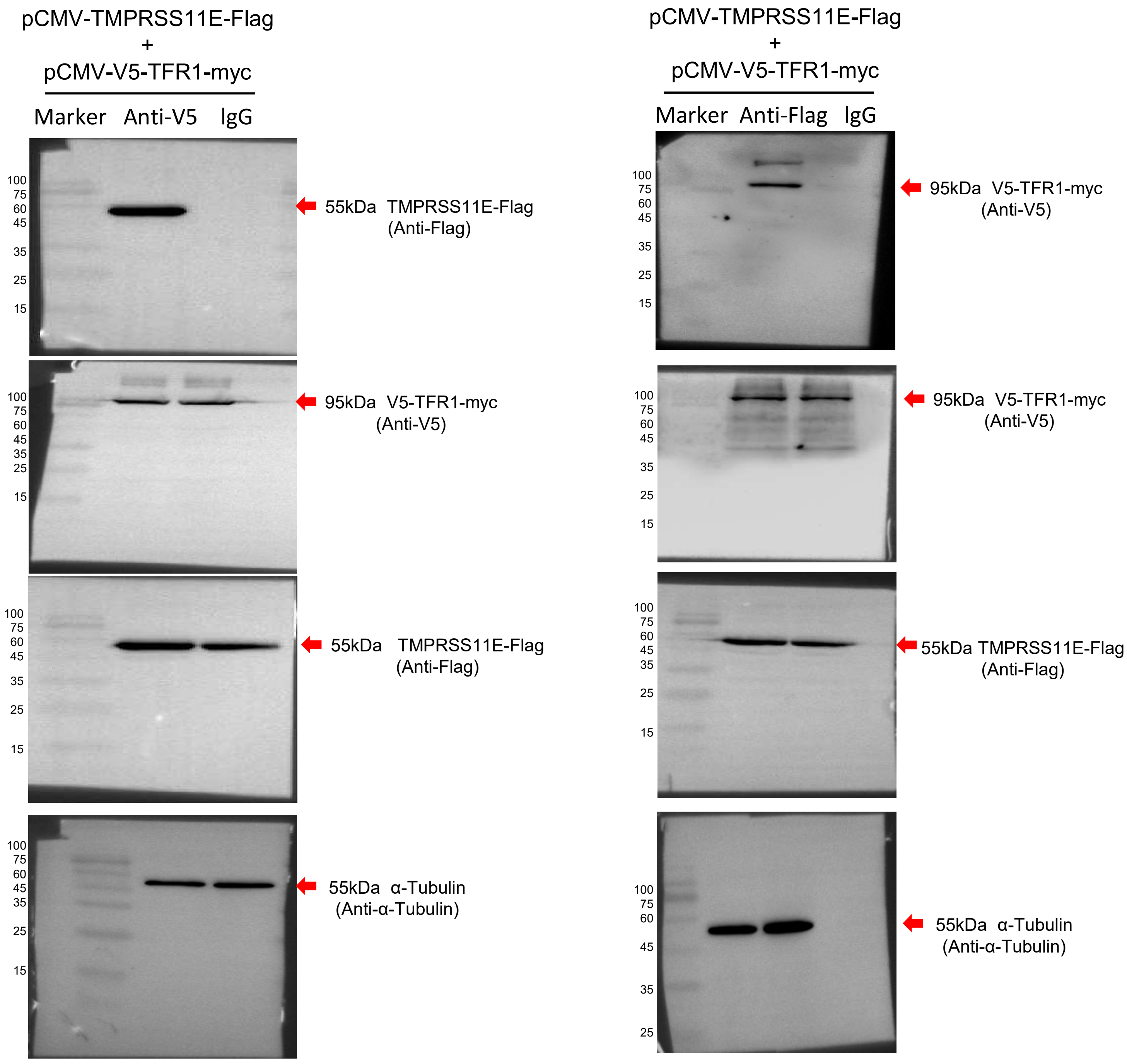

Figure 2A

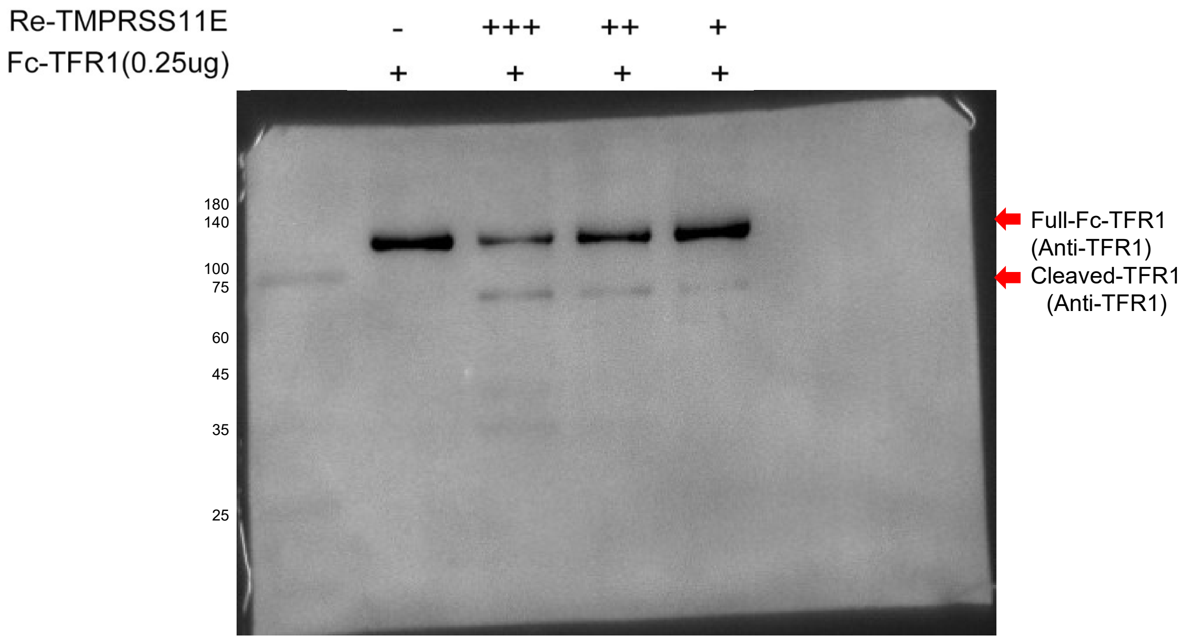
Re-TMPRSS11E - +++ ++ +
Fc-TFR1(0.25ug) + + + +
180
140
100
75
60
45
35
25
Full-Fc-TFR1
(Anti-TFR1)
Cleaved-TFR1
(Anti-TFR1)

Figure 2B

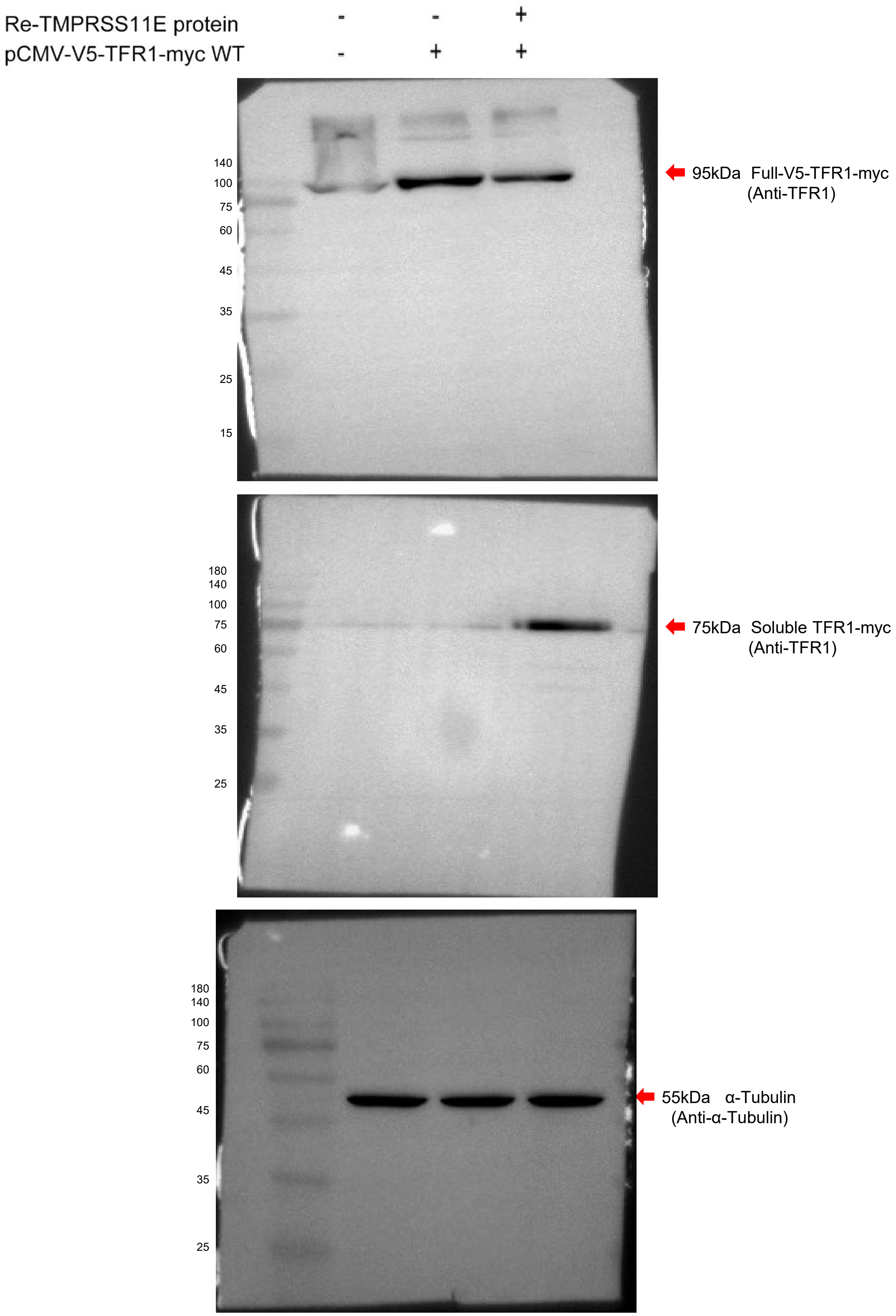
Re-TMPRSS11E protein - - +
pCMV-V5-TFR1-myc WT - + +
140
100
75
60
45
35
25
15
95kDa Full-V5-TFR1-myc
(Anti-TFR1)
180
140
100
75
60
45
35
25
75kDa Soluble TFR1-myc
(Anti-TFR1)
180
140
100
75
60
45
35
25
55kDa α-Tubulin
(Anti-α-Tubulin)

Figure 2C

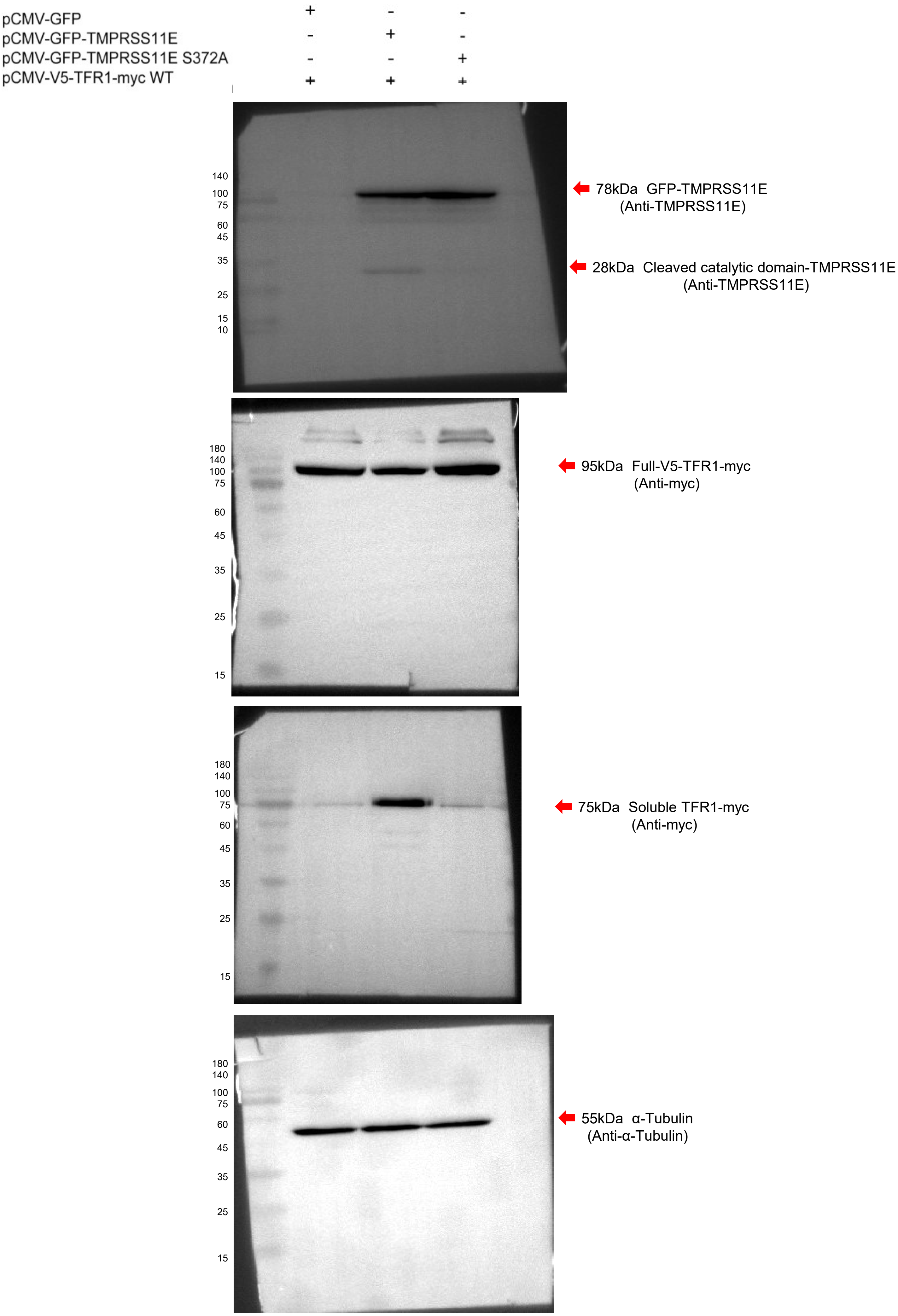

pCMV-GFP + - -
pCMV-GFP-TMPRSS11E - + -
pCMV-GFP-TMPRSS11E S372A - - +
pCMV-V5-TFR1-myc WT + + +
140
100
75
60
45
35
25
15
10
78kDa GFP-TMPRSS11E
(Anti-TMPRSS11E)
28kDa Cleaved catalytic domain-TMPRSS11E
(Anti-TMPRSS11E)
180
140
100
75
60
45
35
25
15
95kDa Full-V5-TFR1-myc
(Anti-myc)
180
140
100
75
60
45
35
25
15
75kDa Soluble TFR1-myc
(Anti-myc)
180
140
100
75
60
45
35
25
15
55kDa α-Tubulin
(Anti-α-Tubulin)

Figure 2D

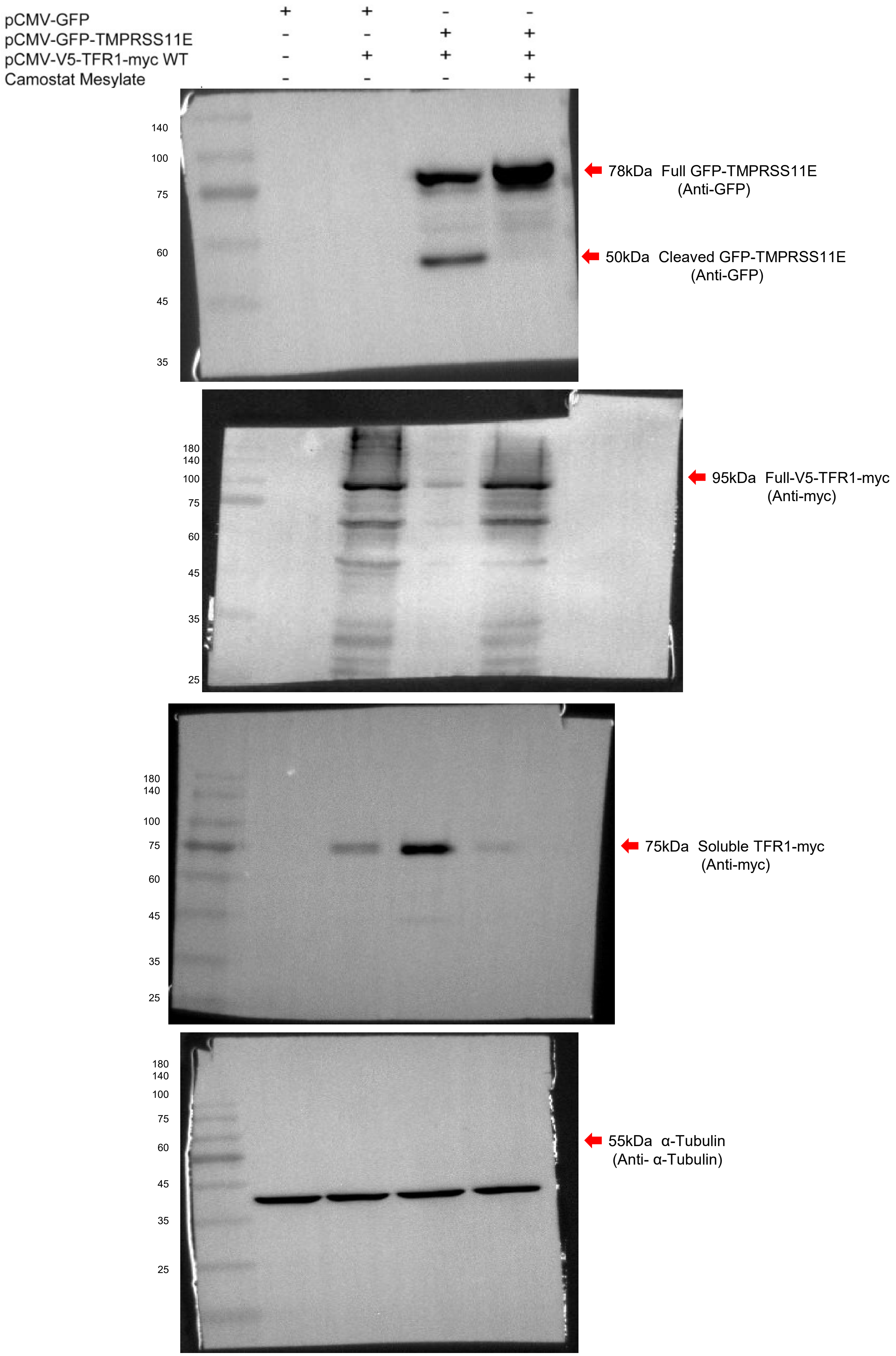

pCMV-GFP + + - -
pCMV-GFP-TMPRSS11E - - + +
pCMV-V5-TFR1-myc WT - + + +
Camostat Mesylate - - - +
140
100
75
60
45
35
78kDa Full GFP-TMPRSS11E (Anti-GFP)
50kDa Cleaved GFP-TMPRSS11E (Anti-GFP)
180
140
100
75
60
45
35
25
95kDa Full-V5-TFR1-myc (Anti-myc)
180
140
100
75
60
45
35
25
75kDa Soluble TFR1-myc (Anti-myc)
180
140
100
75
60
45
35
25
55kDa α-Tubulin (Anti- α-Tubulin)

Figure 3B

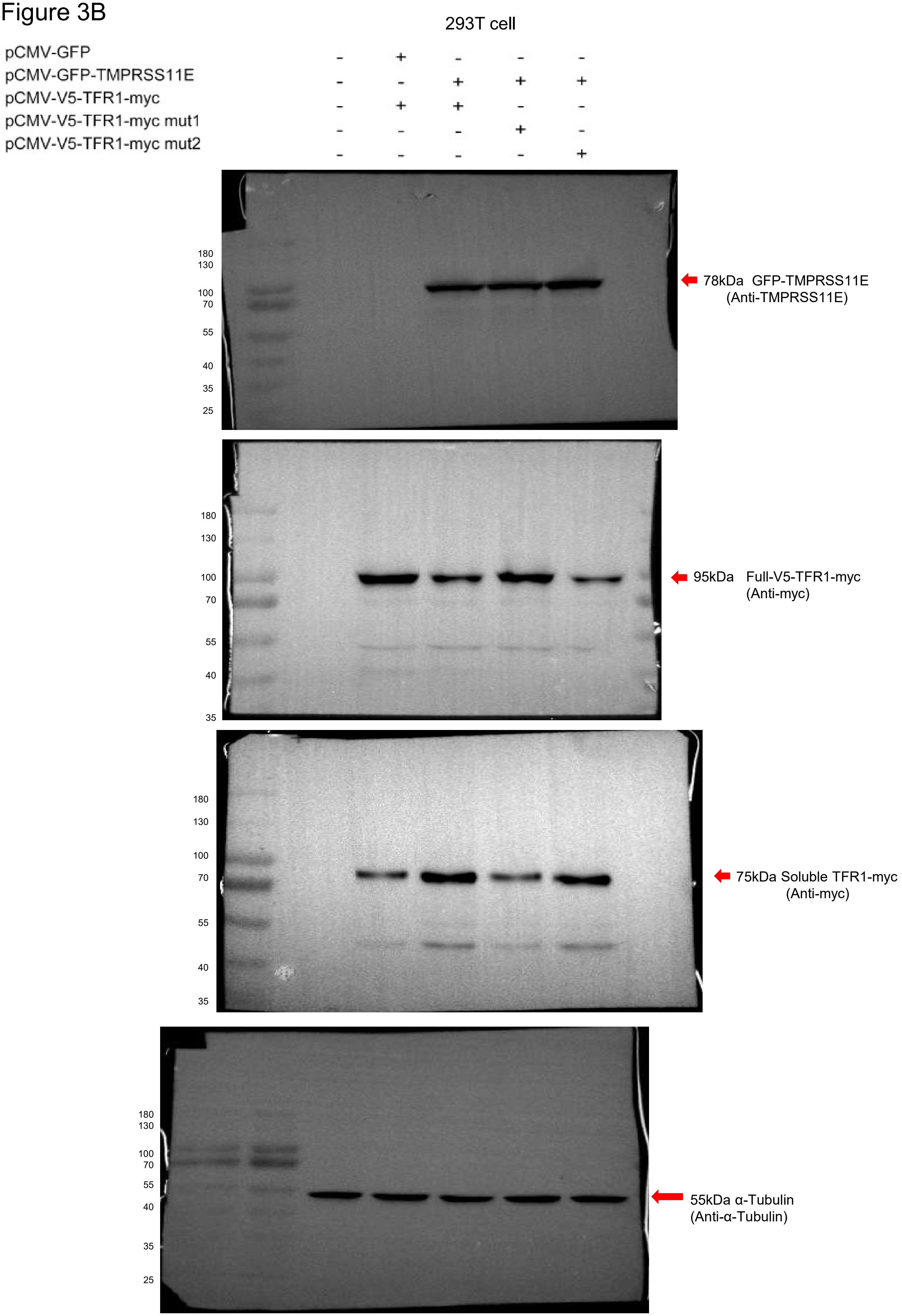

293T cell
pCMV-GFP - + - - -
pCMV-GFP-TMPRSS11E - - + + +
pCMV-V5-TFR1-myc - + + - -
pCMV-V5-TFR1-myc mut1 - - - + -
pCMV-V5-TFR1-myc mut2 - - - - +
180
130
100
70
55
40
35
25
78kDa GFP-TMPRSS11E
(Anti-TMPRSS11E)
95kDa Full-V5-TFR1-myc
(Anti-myc)
75kDa Soluble TFR1-myc
(Anti-myc)
55kDa α-Tubulin
(Anti-α-Tubulin)

Figure 3C

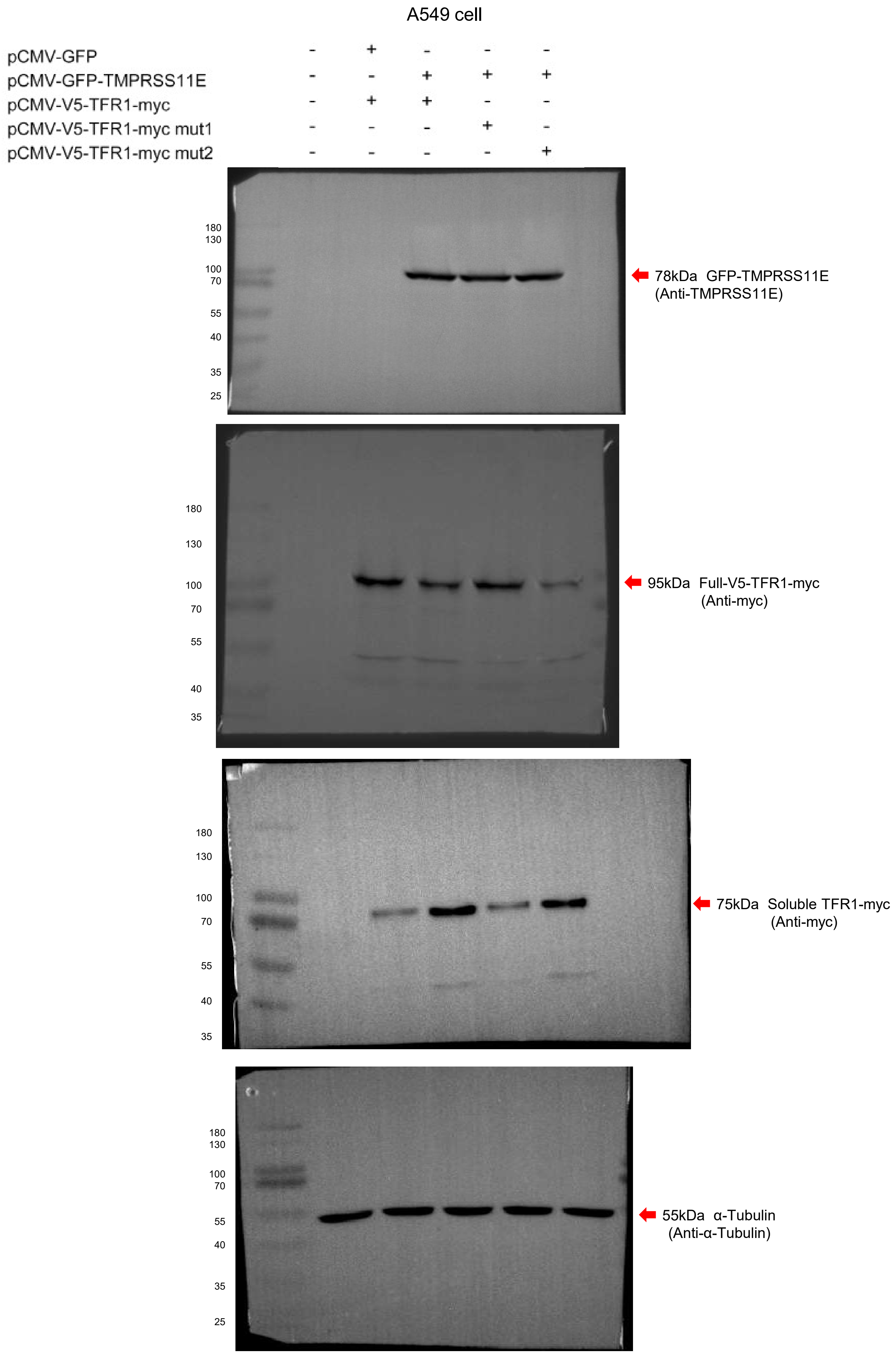
A549 cell
pCMV-GFP - + - - -
pCMV-GFP-TMPRSS11E - - + + +
pCMV-V5-TFR1-myc - + + - -
pCMV-V5-TFR1-myc mut1 - - - + -
pCMV-V5-TFR1-myc mut2 - - - - +
180
130
100
70
55
40
35
25
78kDa GFP-TMPRSS11E
(Anti-TMPRSS11E)
180
130
100
70
55
40
35
95kDa Full-V5-TFR1-myc
(Anti-myc)
180
130
100
70
55
40
35
75kDa Soluble TFR1-myc
(Anti-myc)
180
130
100
70
55
40
35
25
55kDa α-Tubulin
(Anti-α-Tubulin)

Figure 5B

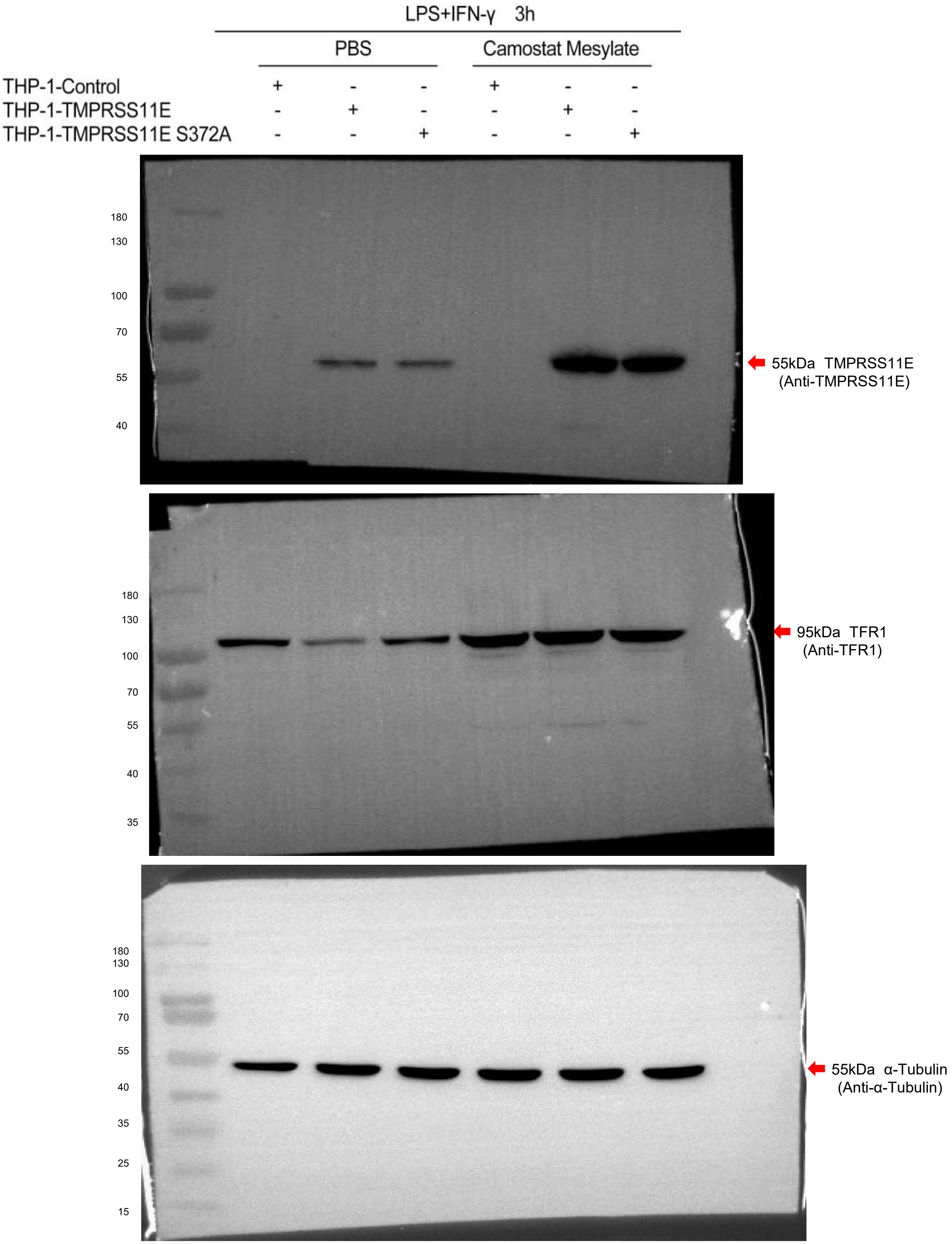
LPS+IFN-γ 3h
PBS
Camostat Mesylate
THP-1-Control + - - + - -
THP-1-TMPRSS11E - + - - + -
THP-1-TMPRSS11E S372A - - + - - +
180
130
100
70
55
40
55kDa TMPRSS11E
(Anti-TMPRSS11E)
180
130
100
70
55
40
35
95kDa TFR1
(Anti-TFR1)
180
130
100
70
55
40
35
25
15
55kDa α-Tubulin
(Anti-α-Tubulin)

Figure 6A3

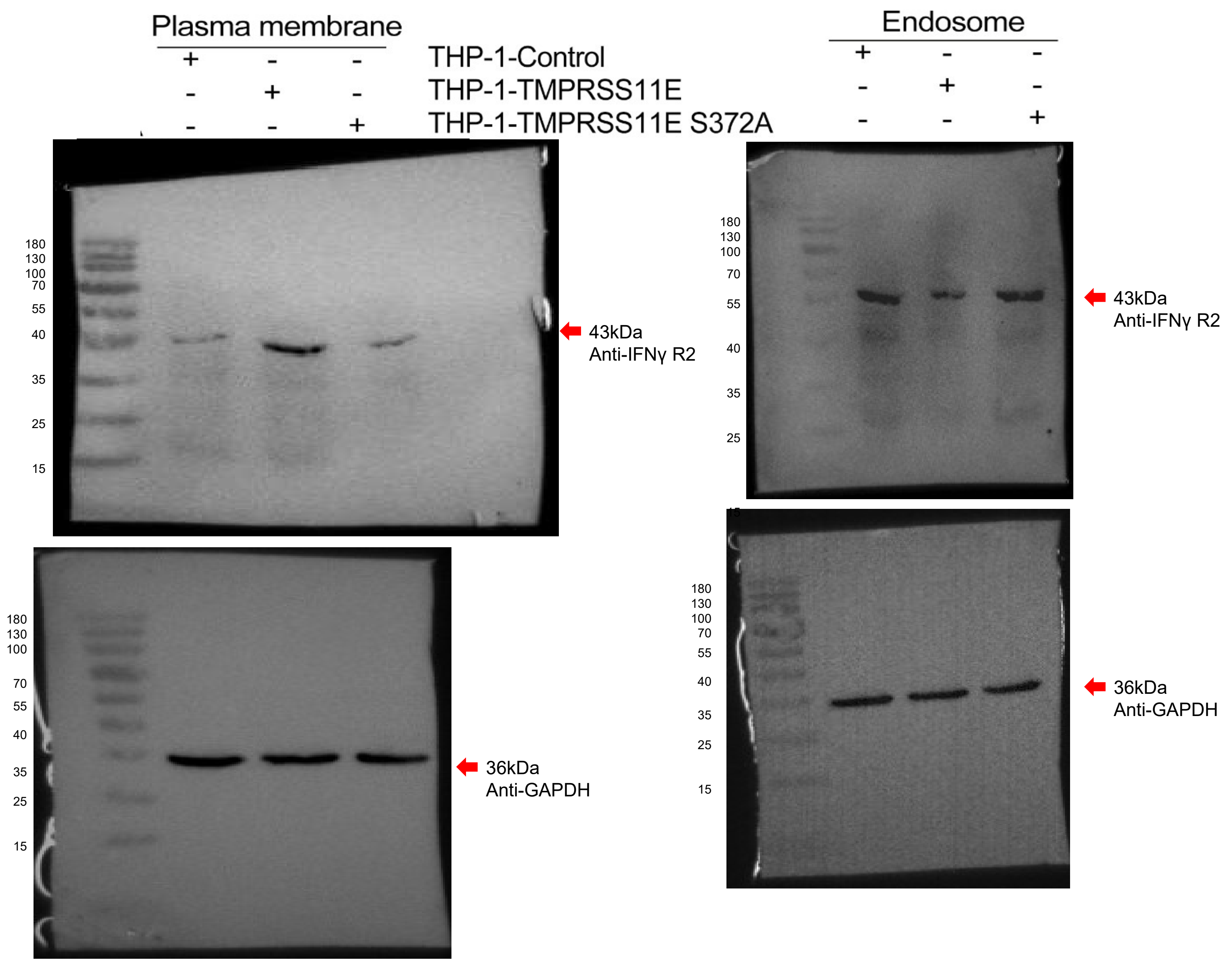
Plasma membrane
Endosome
THP-1-Control
THP-1-TMPRSS11E
THP-1-TMPRSS11E S372A
+ - -
- + -
- - +
180
130
100
70
55
40
35
25
15
43kDa
Anti-IFNγ R2
36kDa
Anti-GAPDH

Figure 6B

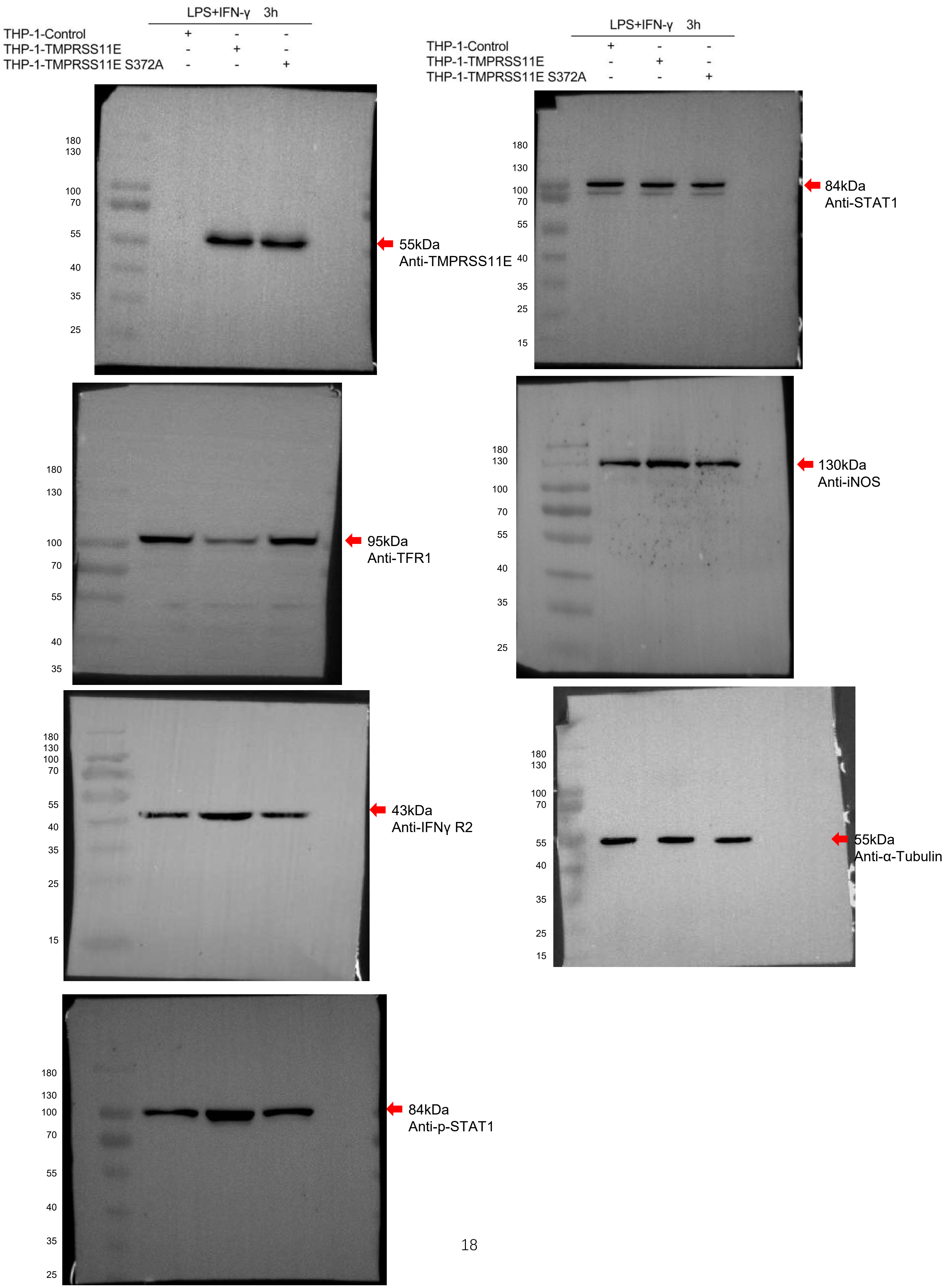
LPS+IFN-γ 3h
THP-1-Control + - -
THP-1-TMPRSS11E - + -
THP-1-TMPRSS11E S372A - - +
55kDa
Anti-TMPRSS11E
84kDa
Anti-STAT1
95kDa
Anti-TFR1
130kDa
Anti-iNOS
43kDa
Anti-IFNγ R2
55kDa
Anti-α-Tubulin
84kDa
Anti-p-STAT1
180
130
100
70
55
40
35
25
15

Figure 7A

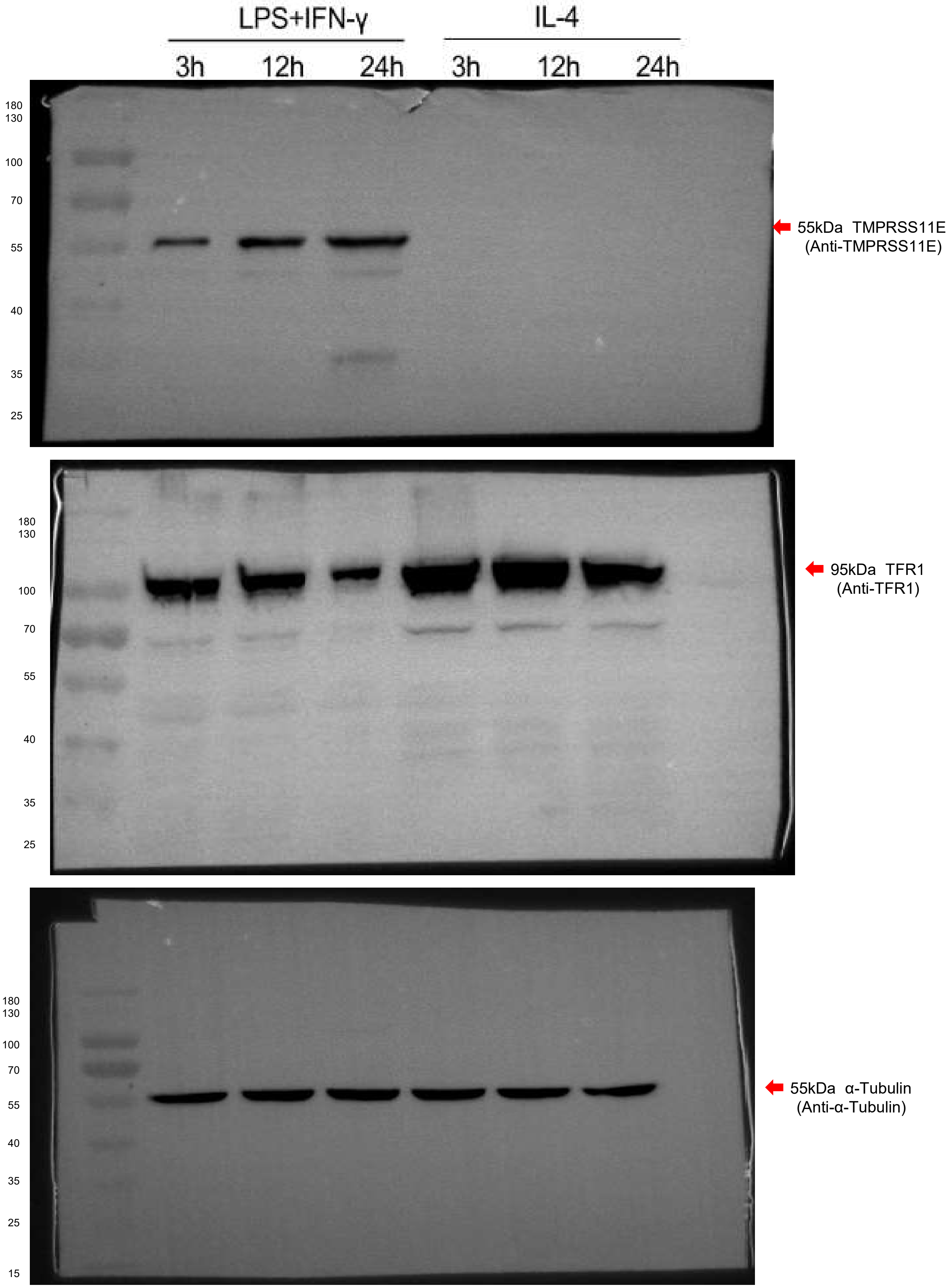

LPS+IFN-γ
IL-4
3h
12h
24h
3h
12h
24h
180
130
100
70
55
40
35
25
55kDa TMPRSS11E
(Anti-TMPRSS11E)
180
130
100
70
55
40
35
25
95kDa TFR1
(Anti-TFR1)
180
130
100
70
55
40
35
25
15
55kDa α-Tubulin
(Anti-α-Tubulin)

Figure 7D

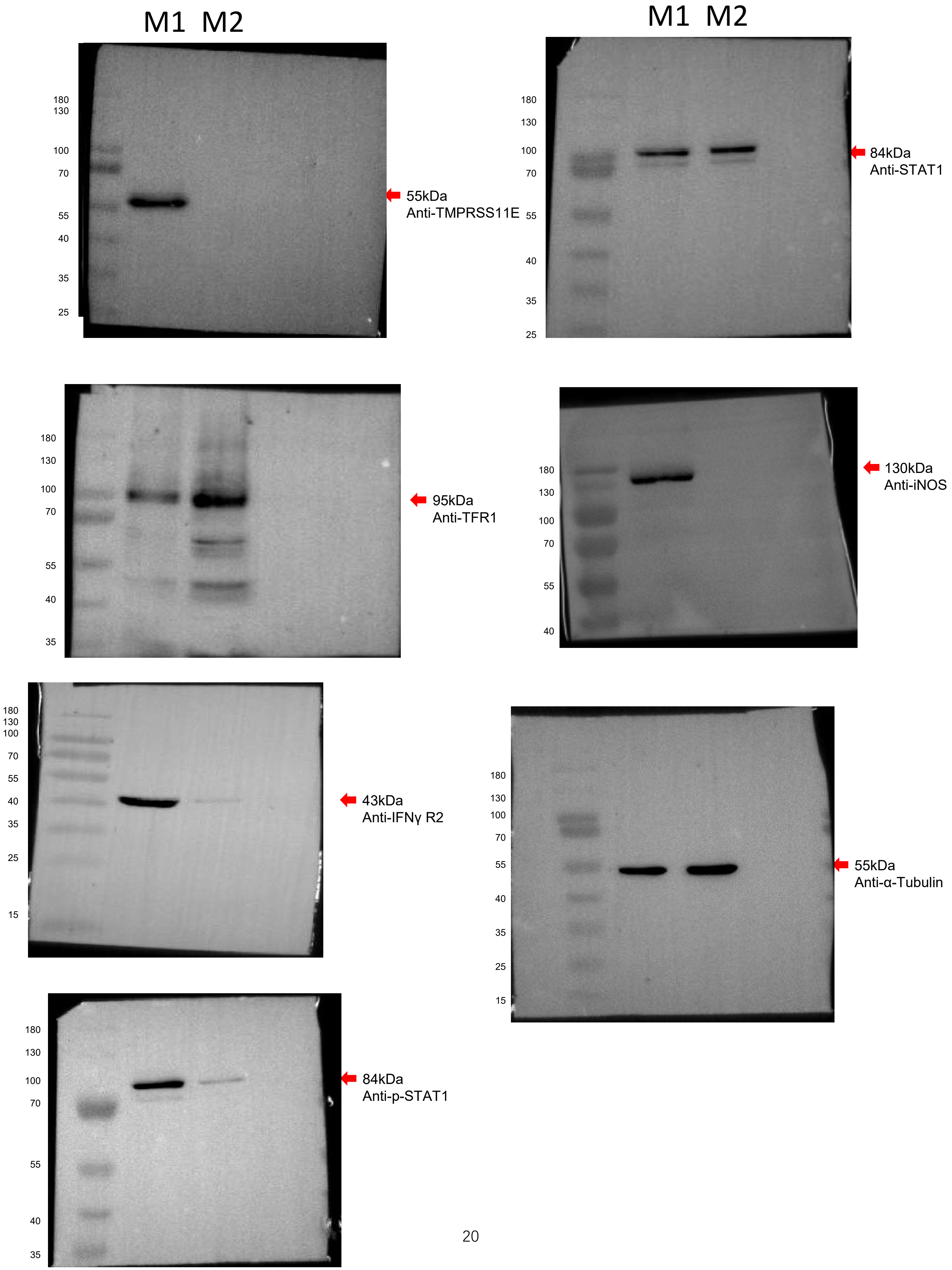
M1 M2
180
130
100
70
55
40
35
25
55kDa
Anti-TMPRSS11E
M1 M2
180
130
100
70
55
40
35
25
84kDa
Anti-STAT1
180
130
100
70
55
40
35
95kDa
Anti-TFR1
180
130
100
70
55
40
130kDa
Anti-iNOS
180
130
100
70
55
40
35
25
15
43kDa
Anti-IFNγ R2
180
130
100
70
55
40
35
25
15
55kDa
Anti-α-Tubulin
180
130
100
70
55
40
35
84kDa
Anti-p-STAT1

Figure 7F

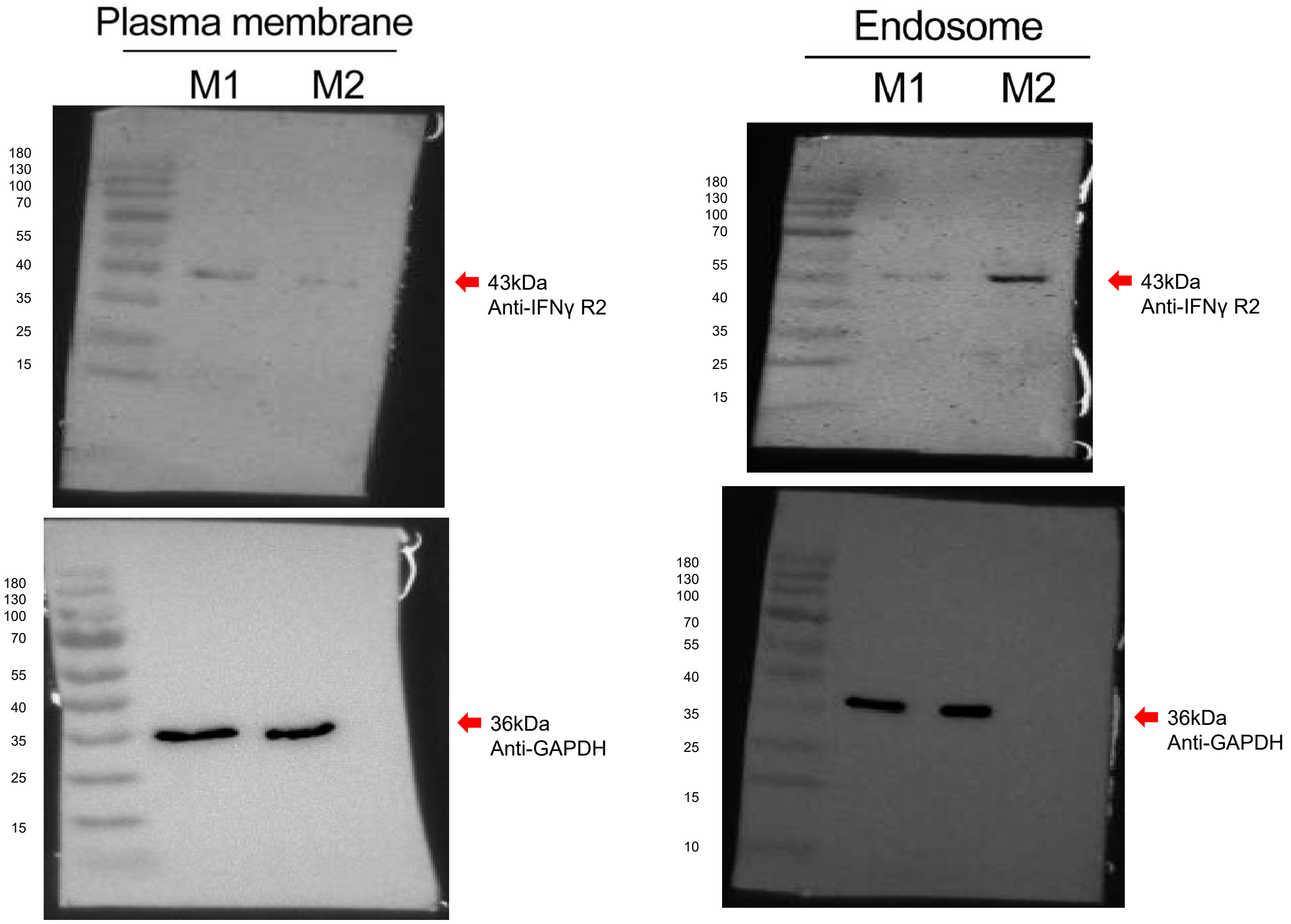
Plasma membrane
M1
M2
180
130
100
70
55
40
35
25
15
43kDa
Anti-IFNγ R2
180
130
100
70
55
40
35
25
15
36kDa
Anti-GAPDH
Endosome
M1
M2
180
130
100
70
55
40
35
25
15
43kDa
Anti-IFNγ R2
180
130
100
70
55
40
35
25
15
10
36kDa
Anti-GAPDH

Figure 8B

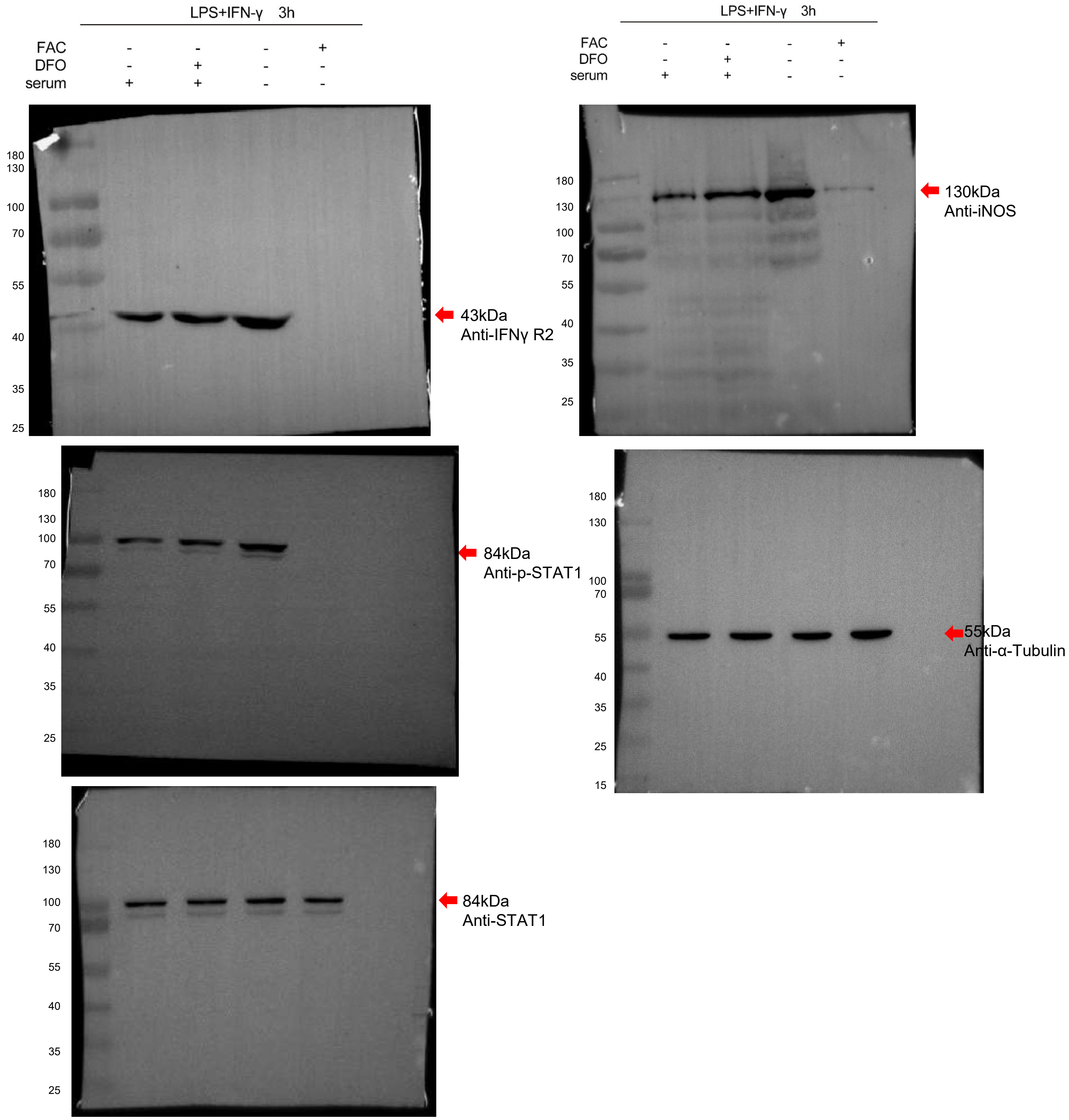

LPS+IFN-γ 3h
FAC - - - +
DFO - + - -
serum + + - -
180
130
100
70
55
40
35
25
43kDa
Anti-IFNγ R2
LPS+IFN-γ 3h
FAC - - - +
DFO - + - -
serum + + - -
180
130
100
70
55
40
35
25
130kDa
Anti-iNOS
180
130
100
70
55
40
35
25
84kDa
Anti-p-STAT1
180
130
100
70
55
40
35
25
15
55kDa
Anti-α-Tubulin
180
130
100
70
55
40
35
25
84kDa
Anti-STAT1

Supplementary Figure 1A

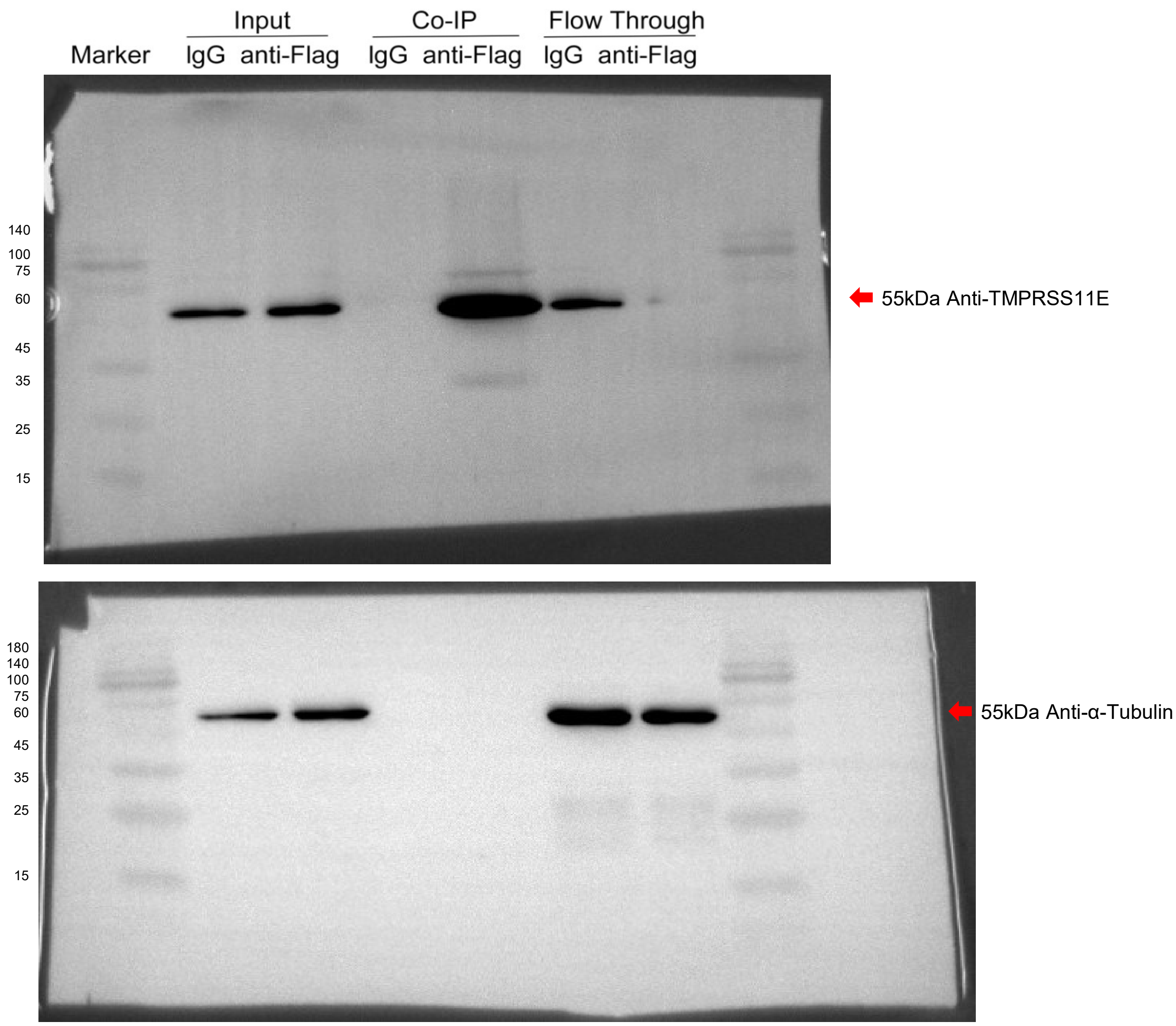

Supplementary Figure 3A

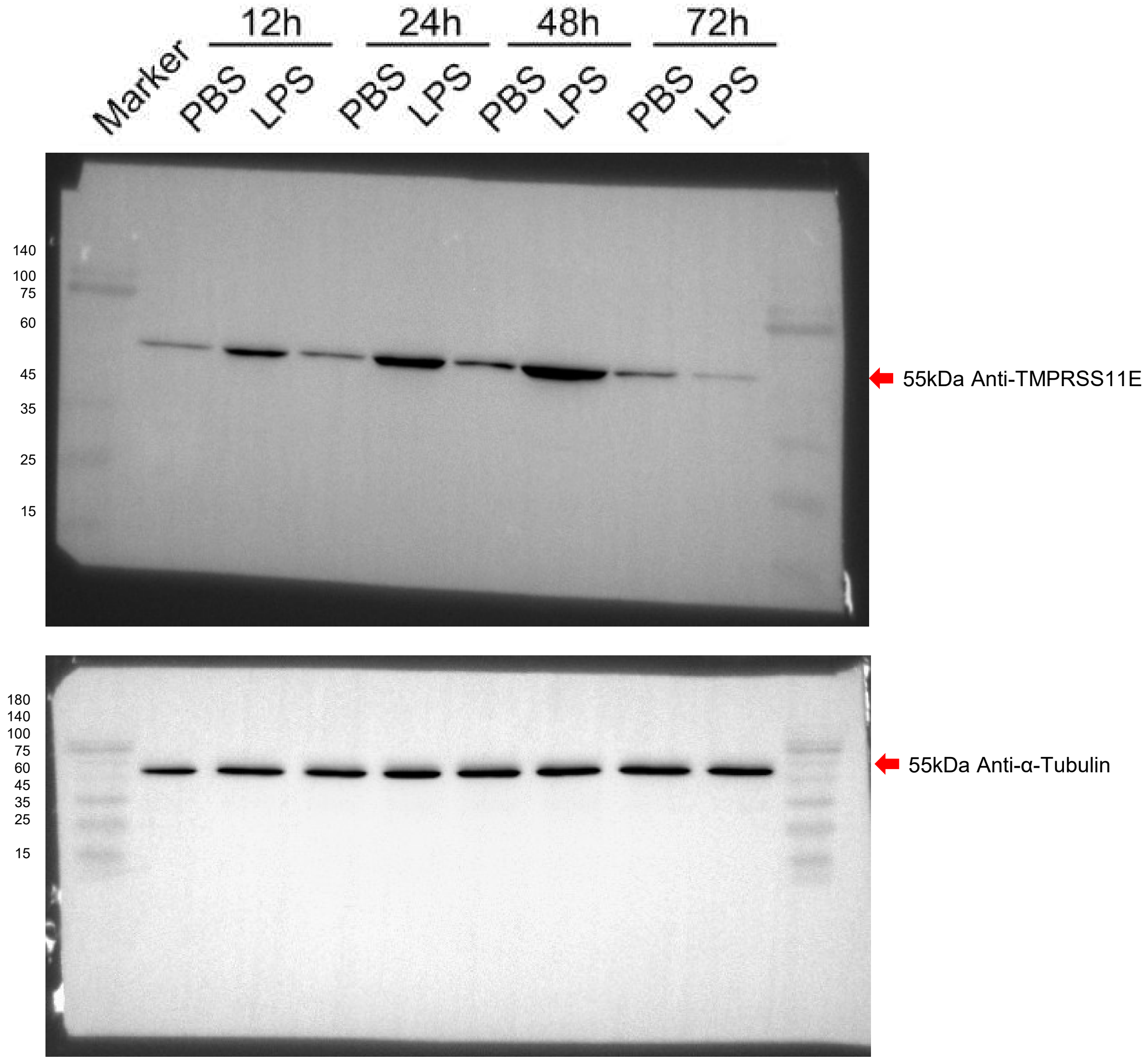

Supplement: Supplementary file 2 — Supplementary Information [file 42003_2025_9132_MOESM2_ESM.pdf]
