# Supplementary material for: TMPRSS11E-mediated TFR1 cleavage influences IFN-γR2 internalization and the macrophage innate response
Source: Commun Biol. 2025 Dec 3;8:1741. doi: 10.1038/s42003-025-09132-2 (PMC12675585; doi:10.1038/s42003-025-09132-2)
Supplement: Supplementary file 3 — Description of Additional Supplementary Files [file 42003_2025_9132_MOESM3_ESM.pdf]

Description of Additional Supplementary Files

File name: Supplementary Data 1

Description: The source data behind the graphs in the paper.

File name: Supplementary Information

Description: Supplementary Figures, Supplementary Tables and Uncropped WB images.
