# Supplementary material for: TMPRSS11E-mediated TFR1 cleavage influences IFN-γR2 internalization and the macrophage innate response
Source: Commun Biol. 2025 Dec 3;8:1741. doi: 10.1038/s42003-025-09132-2 (PMC12675585; doi:10.1038/s42003-025-09132-2)
Supplement: Supplementary file 5 — Reporting summary [file 42003_2025_9132_MOESM5_ESM.pdf]

nature portfolio

Corresponding author(s): Shufeng Li, COMMSBIO-25-1472C

Last updated by author(s): Sep 26, 2025

Reporting Summary

Nature Portfolio wishes to improve the reproducibility of the work that we publish. This form provides structure for consistency and transparency in reporting. For further information on Nature Portfolio policies, see our Editorial Policies and the Editorial Policy Checklist.

Statistics

For all statistical analyses, confirm that the following items are present in the figure legend, table legend, main text, or Methods section.

n/a	Confirmed	
☐	☒	The exact sample size (*n*) for each experimental group/condition, given as a discrete number and unit of measurement
☐	☒	A statement on whether measurements were taken from distinct samples or whether the same sample was measured repeatedly
☐	☒	The statistical test(s) used AND whether they are one- or two-sided<br>*Only common tests should be described solely by name; describe more complex techniques in the Methods section.*
☐	☒	A description of all covariates tested
☐	☒	A description of any assumptions or corrections, such as tests of normality and adjustment for multiple comparisons
☐	☒	A full description of the statistical parameters including central tendency (e.g. means) or other basic estimates (e.g. regression coefficient) AND variation (e.g. standard deviation) or associated estimates of uncertainty (e.g. confidence intervals)
☐	☒	For null hypothesis testing, the test statistic (e.g. *F*, *t*, *r*) with confidence intervals, effect sizes, degrees of freedom and *P* value noted<br>*Give P values as exact values whenever suitable.*
☒	☐	For Bayesian analysis, information on the choice of priors and Markov chain Monte Carlo settings
☒	☐	For hierarchical and complex designs, identification of the appropriate level for tests and full reporting of outcomes
☒	☐	Estimates of effect sizes (e.g. Cohen's *d*, Pearson's *r*), indicating how they were calculated

Our web collection on statistics for biologists contains articles on many of the points above.

Software and code

Policy information about availability of computer code

Data collection	No software was used to collect the data in this study.
Data analysis	The statistical analysis was performed using Student's t test via GraphPad Prism 7.

For manuscripts utilizing custom algorithms or software that are central to the research but not yet described in published literature, software must be made available to editors and reviewers. We strongly encourage code deposition in a community repository (e.g. GitHub). See the Nature Portfolio guidelines for submitting code & software for further information.

Data

Policy information about availability of data

All manuscripts must include a data availability statement. This statement should provide the following information, where applicable:
- Accession codes, unique identifiers, or web links for publicly available datasets
- A description of any restrictions on data availability
- For clinical datasets or third party data, please ensure that the statement adheres to our policy

All data supporting this study are presented in this manuscript.

Research involving human participants, their data, or biological material

Policy information about studies with human participants or human data. See also policy information about sex, gender (identity/presentation), and sexual orientation and race, ethnicity and racism.

Reporting on sex and gender	Clinical samples were collected from Southeast University Affiliated Zhongda Hospital. A total of 20 pneumonia patients and 10 control patients were included. The relevant data are provided in Supplementary Material. Gender was not considered as a primary influencing factor in this study.
Reporting on race, ethnicity, or other socially relevant groupings	In our manuscript, we did not utilize any socially constructed or socially relevant categorization variables such as race, ethnicity, socioeconomic status, or gender identity. The focus of this study was primarily on the clinical characteristics related to pneumonia, and we did not incorporate these variables into our analysis.
Population characteristics	The study did not specifically focus on or collect data regarding covariate-relevant population characteristics such as age, genotypic information.
Recruitment	A total of 20 patients with acute lung inflammation and 10 control individuals were recruited for clinical analysis at Zhongda Hospital, Southeast University, China. Ten patients who were admitted for the evaluation of solitary pulmonary nodules (SPNs) without evidence of pulmonary infection composed the control group. Twenty patients was diagnosed as acute lung inflammation without treatment.
Ethics oversight	The study was approved by the Ethics Committee of the Zhongda Hospital.

Note that full information on the approval of the study protocol must also be provided in the manuscript.

Field-specific reporting

Please select the one below that is the best fit for your research. If you are not sure, read the appropriate sections before making your selection.

☒ Life sciences ☐ Behavioural & social sciences ☐ Ecological, evolutionary & environmental sciences

For a reference copy of the document with all sections, see nature.com/documents/nr-reporting-summary-flat.pdf

Life sciences study design

All studies must disclose on these points even when the disclosure is negative.

Sample size	The sample size number was determined to balance the need for sufficient statistical significance and ethical principles of minimizing animal use.
Data exclusions	No data were excluded from the analyses.
Replication	All attempts at replication were successful.
Randomization	Animals and cells were randomly allocated into experimental groups.
Blinding	Investigators were blinded to group allocation during both data collection and analysis.

Reporting for specific materials, systems and methods

We require information from authors about some types of materials, experimental systems and methods used in many studies. Here, indicate whether each material, system or method listed is relevant to your study. If you are not sure if a list item applies to your research, read the appropriate section before selecting a response.

Materials & experimental systems

n/a	Involved in the study	
☐	☒	Antibodies
☐	☒	Eukaryotic cell lines
☒	☐	Palaeontology and archaeology
☐	☒	Animals and other organisms
☒	☐	Clinical data
☒	☐	Dual use research of concern
☒	☐	Plants

Methods

n/a	Involved in the study	
☒	☐	ChIP-seq
☐	☒	Flow cytometry
☒	☐	MRI-based neuroimaging

Antibodies

Antibodies used	All antibodies used in the study are shown in Supplementary Material Table S1: “Plasmids and Antibodies.”
Validation	The application of the antibodies were according to the manufacturer's instructions.

Eukaryotic cell lines

Policy information about cell lines and Sex and Gender in Research

Cell line source(s)	The source of cell lines were shown in methods.
Authentication	All the cell lines were authenticated using a short tandem repeat DNA profiling.
Mycoplasma contamination	All cell lines are negative for Mycoplasma contamination.
Commonly misidentified lines (See ICLAC register)	NA

Animals and other research organisms

Policy information about studies involving animals; ARRIVE guidelines recommended for reporting animal research, and Sex and Gender in Research

Laboratory animals	Male C57BL/6 mice (aged 6–8 weeks) or male SD rats (aged 6–7 weeks) were purchased from Model Animal Research Center (MARC; Nanjing, China).
Wild animals	NA
Reporting on sex	Sex was not considered as a primary influencing factor in this study.
Field-collected samples	This study did not involve samples collected from the field.
Ethics oversight	The experimental protocol for animals was approved by Animal Experimentation Ethics Committee of Southeast University.

Note that full information on the approval of the study protocol must also be provided in the manuscript.

Plants

Seed stocks	NA
Novel plant genotypes	NA
Authentication	NA

Flow Cytometry

Plots

Confirm that:

- [x] The axis labels state the marker and fluorochrome used (e.g. CD4-FITC).
- [x] The axis scales are clearly visible. Include numbers along axes only for bottom left plot of group (a 'group' is an analysis of identical markers).
- [x] All plots are contour plots with outliers or pseudocolor plots.
- [x] A numerical value for number of cells or percentage (with statistics) is provided.

Methodology

Sample preparation	Detailed information was described in the manuscript.

Instrument	Cytek Northern lights
Software	Flowjo
Cell population abundance	Total amount of 10000 cells per time.
Gating strategy	Use the blank to set the voltage, then select the main cell group, choose the gating and then select FSC/SSC gates to remove debris, use Single Parameter Histograms to show the percentage of positive-stained cells.

☒ Tick this box to confirm that a figure exemplifying the gating strategy is provided in the Supplementary Information.
